# Emulsifying Stability, Digestive Sustained Release, and Cellular Uptake of Alcohol-Soluble *Artemisia argyi* Flavonoids Were Improved by Glycosylation of Casein Micelles with Oat Glucan

**DOI:** 10.3390/foods14142435

**Published:** 2025-07-10

**Authors:** Ye Zhang, Dongliang Wang, Mengling Peng, Min Yang, Ya Yu, Mengting Yuan, Yanan Liu, Bingyu Zhu, Xiuheng Xue, Juhua Wang

**Affiliations:** 1 College of Veterinary Medicine, Anhui Agricultural University, Hefei 230036, China; 18856066194@163.com (Y.Z.); menglingpeng@ahau.edu.cn (M.P.); yangabd36@163.com (M.Y.); 17638359580@163.com (B.Z.); 2 School of Horticulture, Anhui Agricultural University, Hefei 230036, China; wangdongliang@ahau.edu.cn; 3 School of Food and Nutrition, Anhui Agricultural University, Hefei 230036, China; 19855278715@163.com (Y.Y.); 19556901509@163.com (M.Y.); 15329837902@163.com (Y.L.)

**Keywords:** *Artemisia argyi* flavonoids, glycosylation reaction, casein micelles, emulsifying stability, digestive sustained release

## Abstract

Flavonoids, widely present in *Artemisia argyi* (AA), offer potential health benefits but are limited in food applications because of their bitter taste, inadequate absorption, and stability. Casein micelles encapsulation can enhance the flavonoid absorption, stability, and bioactivity. In this study, *Artemisia argyi* flavonoids (AAFs) were extracted using ultrasound-assisted extraction (UAE) to optimize the process. The glycosylation reaction between casein (CN) micelles and oat β-glucan (OBG) was employed to improve AAF’s emulsifying stability, sustained release during digestion, and cellular uptake. The maximum glycosylation degree of 32.33% was achieved at a CN-to-OBG ratio of 1:2, 120 min browning time, and 95 °C temperature. This glycosylated delivery system enhanced the emulsifying properties of the AAFs, digestive sustained release, and cellular uptake, showing potential as a cross-linking material for fat-soluble substances and medicines.

## 1. Introduction

*Artemisia argyi* (AA), a commonly used herbaceous plant in the Artemisia genus of the Asteraceae family with a high economic value, has been used for a long time and is widely distributed in many regions of the world [1]. In addition to its nutritional value, AA contains abundant active ingredients and special metabolites. Many bioactive compounds, particularly flavonoids, are plant secondary metabolites that are readily soluble in alcohol because of their diverse structures [1]. Flavonoids are increasingly attracting scientists’ attention because of their inflammation-reducing, oxidation-resisting, and microbe-killing properties with their potential nutritional and medicinal applications [2]. Therefore, it is highly desirable to extract and utilize the abundant flavonoids in AA. Consequently, the extraction of bioactive components from natural sources has gained significant attention in recent years, driven by the growing demand for natural and functional ingredients in food [3]. However, poor water solubility, unpleasant taste, low absorption rate, and instability greatly limit their application in the food. Furthermore, the gastrointestinal stability and absorption of flavonoids might be affected by their lipid solubility. Thus, it is necessary to adopt the proper processing methods to regulate the antioxidant, digestive, sustained release, and cellular delivery of *Artemisia argyi* flavonoids (AAF) for their more extensive application in food industry. Microencapsulation and microemulsion technologies, combined with the addition of emulsifying agents such as pectin, protein, and polysaccharides, can enhance the emulsification, oxidation stability, and delivery of flavonoids [4].

Micelle systems have a hydrophobic core and a hydrophilic surface, which provide excellent stability in aqueous solutions and can deliver a large amount of nutrition components and medicines while sustaining their release [5]. Casein (CN) is the most abundant protein in milk accounting for about 80%, consisting of α_s1_-casein, α_s2_-casein, β-casein, and κ-casein, which spontaneously forms core-shell micelles with a diameter of about 50–500 nm through hydrophobic interaction, electrostatic interaction, and hydrogen bonding [6]. In the existing research, the nanocluster model holds that the interior of casein micelles is formed by the interconnection of calcium phosphate and casein molecules to create a micro-cluster complex structure. The hydrophilic end of κ-casein on the surface of the micelles extends outward from the surface, β-casein is located inside the micelles, and two types of αs-casein are distributed throughout the micelle structure [7]. CN has an open rheological structure that can be reassembled into micellar structures to capture target molecules, which can interact with protein molecules through hydrophobic interactions, and then be assembled into casein nanoparticles [8]. CN micelles have been shown to be a potential self-aggregating encapsulation agent for fat-soluble substances, and their shape and size are unaffected by the presence of an oil phase [9]. Moreover, the nanocarriers of bioactive-loaded casein micelles play a crucial role in influencing colloidal stability, release rate, and intestinal wall delivery [10]. The size and charge of bioactive-loaded nanocarriers influence their gastrointestinal uptake via tight junctions, influx transporters, and passive transport mechanisms [11]. As a natural biomaterial, CN micelles are regarded as an excellent encapsulation agent and delivery system for fat soluble substances and drugs due to their excellent emulsion capacity and high surface activity [12]. Due to its intermolecular hydrophobic and amphiphilic structure, CN can self-assemble into micelles in aqueous solutions, making β-casein an ideal delivery system for fat-soluble substances [13]. However, native caseins are thermally unstable, and their large structure is not conducive to the delivery and cellular uptake of nutrition components and medicines. Recent studies have employed the Maillard reaction (MR) between whey protein and oligosaccharides to significantly enhance the embedding rate and bio-accessibility of lycopene [14]. Since the MR is a mild process that does not necessitate the incorporation of outside chemicals, it is considered an environmentally friendly, effective, and dependable method to enhance the functionality of food proteins.

Glycosylation, also known as the MR, is a reaction between the carbonyl group of a reducing sugar and the amino group of an amino acid residue of a polypeptide or protein and has been recognized as an effective and promising method of protein modification to improve the functional activity of proteins or polypeptides in vitro and in vivo [15,16]. There are many discrepancies in the results of the current studies on the changes in emulsification activity and emulsion stability after the glycosylation of casein. It was found that the emulsification activity of glucose-casein was improved over the single casein emulsification activity and the emulsification stability could be improved [17]. Zhang et al. [18] showed that after glycosylation with chitosan significantly improved the encapsulation of quercetin by casein, while also maintaining or improving the stability and water solubility of quercetin. Chang et al. [19] found that pectin–casein-loaded curcumin up to 96% can achieve a slow release of curcumin, as well as a significant increase in the bioavailability of curcumin.

Oat β-glucan (OBG), a major component of oat soluble fiber composed of β-(1-3) and β-(1-4) bonds in a linear branched chain of β-D-glucopyranosyl molecules, possesses excellent physical and chemical properties such as heat, acid, and alkali resistance, high viscosity, and good gel properties [20], making it a suitable food stabilizer or thickener. Additionally, it can help reduce blood cholesterol, improve intestinal flora composition, exert an anti-tumor effect, and prevent type II diabetes [21]. Zheng et al. [22] verified that adding the isolated whey protein complex and OBG obtained after heat treatment as a thickener to yogurt could enhance the structural and sensory characteristics of yogurt. Studies have shown that the addition of OBG to yogurt could improve the survival rate of *Bifidobacterium longum* at 4 °C, increase the flavor and organoleptic quality of yogurt, and improve the quality stability during refrigerated storage [23]. It is generally believed that the addition of OBG can not only improve the taste of food but also increase the added value of food and provide more possibilities for food processing.

Ultrasonic-assisted extraction (UAE), as a non-thermal food processing technology, is gaining wide potential in the food industry. Low frequency (20–100 kHz) operation generates intense physical effects like shear, turbulence, and the number of radicals [24]. Researchers have demonstrated that ultrasonic treatment loosens the compact structure of casein micelles, reduces their particle size, increases their specific surface area, and enhances their flexibility, thereby improving the solubility of micellar casein powder [25]. Additionally, ultrasonic cavitation induces modifications in the size and shape of CN micelles and fat spheres, potentially forming new fat–casein complexes and enhancing their physicochemical properties [26]. Therefore, the extraction process of AAFs using UAE was first optimized using response surface design, and glycosylation casein micelles were self-assembled by using the glycosylation reaction of CN micelles with OBG to improve the emulsifying stability, digestive sustained release, and cellular uptake of flavonoids. The mechanism of using a glycosylation reaction to enhance the stability of CN micelles was explored. The effects of glycosylation CN micelles on the size of the particles, morphology, and the flavonoids encapsulation efficiency were subsequently determined. Furthermore, the release profile in vitro and cellular uptake of flavonoids loaded with glycosylation CN micelles was investigated. The application and development potential of this system in the food industry will be improved.

## 2. Materials and Methods

### 2.1. Materials

OBG (Penghua Biotechnology Co., Ltd., Xi’an, China), CN and trypsin (Guoyao Reagent Co., Ltd., Beijing, China), and an intestinal epithelial cell line (Shanghai Institute for Biological Sciences, Shanghai, China) were obtained. The reagents including O-phthalaldehyde (OPA) solution, phosphate-buffered solution (PBS), calcium chloride, sodium hydroxide, monobasic potassium phosphate, sodium chloride, hydrogen chloride, ammonium carbonate, sodium bicarbonate, and magnesium chloride were procured from Guosheng Reagent Co., Ltd. (Shanghai, China). Sigma Chemical Co. (St. Louis, MO, USA) was the supplier for the pancreatin, Nile Red, DAPI, and Dulbecco’s Modified Eagle’s Medium (DMEM). In this study, all chemical reagents and solvents utilized were of analytical grade.

### 2.2. Extraction and Identification of Total Flavonoids from AA

An accurately weighed AA powder (1.0 g) was separately blended with 30 mL of ethanol solution in a 50 mL tube. Each mixture was then subjected to UAE. After extraction, following centrifugation of the mixtures under 7500 rpm for 15 min, the supernatant was transferred to a 200 mL volumetric flask. The residue was extracted twice more, and the extraction solutions were combined to yield the total flavonoids of AA. The effects of several ultrasonic-assisted extraction factors, including ultrasonic intensity (100, 200, 300, 400, and 500 W), liquid-to-solid ratio (20, 25, 30, 35, and 40 mL/g), and ethanol concentration (30, 40, 50, 60, 70%) on the yield of total flavonoids from AA were studied. Experiments were designed using the Box–Behnken design (BBD) within the framework of response surface methodology (RSM), which included four factors and three levels, using Design-Expert 12 software (Table S1).

The extracted flavonoids from AA were purified using AB-8 macroporous resin (Beijing Solaibao Technology Co., Ltd., Beijing, China) at a flow velocity of 1.0 mL/min. Flavonoid content was determined via high-performance liquid chromatography–tandem mass spectrometry (HPLC/MS; UHPLC-X500R, AB SCIEX, Framingham, MA, USA). To outline changes in total flavonoids from AA under different formulations and to identify differential flavonoids, hierarchical cluster analysis (HCA) and principal component analysis (PCA) were conducted after normalizing the data using the metabolome package in R (version 3.6.3).

### 2.3. Fabrication of Glycosylation CN Micelles by OBG

Preparation of CN solution: accurately weigh and dissolve the quantitative CN in 0.2 mol/L phosphate-buffered solution (pH 7.4), stir magnetically for 2–2.5 h at room temperature to fully dissolve it into a homogeneous CN solution with a concentration of 6%, and then store it at 4 °C for subsequent use. After mixing and dissolving the CN solution with OBG in a certain mass ratio, it was placed in a constant temperature water bath and heated and stirred. CN and OBG after ultrasonic treatment were mixed, fully stirred, and dissolved, and the sample was next subjected to a water bath, then quickly removed from the ice bath to cool for reaction termination.

Moist heat glycosylation was used to study the effects of the addition ratio of CN and OBG, browning temperature, and time on the degree of glycation reaction. The effects of different CN-to-oat β-glucan mass ratios (1:1, 1:1.5, 1:2, 1:2.5, and 1:3), browning time (60, 90, 120, 150, and 180 min), and browning temperature (80, 85, 90, 95, and 100 °C) on the degree of glycosylation reaction were studied. The impact of ultrasonic power on casein glycosylation was investigated using an ultrasonic cell crusher (20 kHz) at constant power levels of 100, 200, 300, 400, and 500 W for 5 min, with pulse durations of 5.0 s on and 5.0 s off.

### 2.4. AAFs Loaded by Glycosylation CN Micelles

AAF-loaded CN micelles were prepared by mixing an AAF phosphate-buffered solution with micelle solutions at various ratios (1:1, 2:1, 4:1, 6:1, and 8:1) under magnetic stirring for 2 h. The mixture, after pH adjustment to 7.0, was continuously stirred on a magnetic stirrer for 3 h. Then the solution was centrifuged at 3000× *g* for 10 min, and the AAF emulsion encapsulated by methylcellulose (MC) was collected. To determine the total AAF, 2 g of the sample was dissolved in 15 mL of distilled water at 60 °C. After introducing 25 mL of a 3:1 (*v*/*v*) hexane/isopropanol solvent mixture into the solution, the mixture was vortexed for 20 min and then centrifuged at 10,000× *g* for 20 min. The solution underwent centrifugation at 10,000× *g* for 20 min. The resulting supernatant was re-extracted using the same solvent mixture and subsequently filtered. The combined filtrate was then evaporated at 70 °C with a rotary evaporator. In the final step, the AAFs were dried at 105 °C until a constant weight was reached. For comparison, distilled water served as the control, and each sample was analyzed in triplicate. Load rate (%) were calculated using Equation (1).

(1) Load rate (%) = (Amount of embedded AAF/Total amount of AAF) × 100

### 2.5. The Binding Capacity of CN with Oat β-Glucan

#### 2.5.1. Determination of Browning Index of Glycosylation Reaction

Take an appropriate amount of the sample supernatant obtained by centrifugation after the reaction, dilute it 10-fold with a dilution of 0.1% SDS, use the dilution solution as a blank control, and measure the absorbance value at 420 nm of wavelength. For each group, the browning index increased with higher absorbance values, and three parallel measurements were taken.

#### 2.5.2. Determination of Grafting Degree

For the preparation of the AAF reagent, 40.0 mg of AAFs was first dissolved in methanol (1.0 mL). Next, the mixture was prepared by adding 25.0 mL of 0.1 mol/L borax, 2.5 mL of 20% (*w*/*w*) SDS, and 100 μL of β-mercaptoethanol, followed by thorough mixing. Lastly, the mixture was brought to a final volume of 50 mL with distilled water in a volumetric flask.

Dilute the sample to 2 mg/mL with the phosphate buffer, add 4 mL of AAF reagent to 200 μL of 2 mg/mL sample solution, mix well, react in a 35 °C constant temperature water bath for 2 min, measure the absorbance with a UV spectrophotometer at 340 nm wavelength, and use OPA reagent as a blank control to calculate the grafting degree (DG, %) (2).

(2) DG (%) = A_0_ – A_1_/A_0_ × 100

where A_1_—absorbance of the glycosylated product solution and A_0_—the absorbance of the original protein under the same reaction conditions.

#### 2.5.3. Fourier Transform Infrared (FTIR) Analysis

A VERTEX80 FT-IR spectrometer (Bruker Optik GmbH, Ettlingen, Germany) was used to record spectra at 4 cm^−1^ resolution (400–4000 cm^−1^, room temperature). Conformational changes of CN were examined by analyzing normalized second derivative spectra with Peakfit software (Version 4.12, Systat Software Inc., San Jose, CA, USA).

#### 2.5.4. Analysis by X-Ray Diffraction (XRD)

The samples’ X-ray diffractograms were generated by a SmartLab SE diffractometer (Rigaku Corporation, Tokyo, Japan) under 40 kV/100 mA. In a 2θ range of 5–55°, diffractograms and measurements were taken at a scanning rate of 2°/min.

#### 2.5.5. Analysis by Transmission Electron Microscopy (TEM)

An HT7700 TEM (Hitachi, Tokyo, Japan) with an 80 kV acceleration voltage was used to observe the morphology of the micelles. The sample was first diluted, and then 20 μL of it was placed on a copper mesh. Afterward, the sample on the mesh was stained with a 1% phosphotungstic acid solution for 3 to 5 s. The copper mesh was allowed to dry naturally before being examined with the TEM.

### 2.6. Characterization of AAFs Loaded by CN and CN-OBG Micelles

#### 2.6.1. The Activity and Stability of Emulsification

The turbidity method was employed to analyze the emulsifying activity (EAI) and emulsifying stability (ESI) [27]. In brief, 10 mL of OBG-AAF, CN-AAF, and CN-OBG-AAF micelle solutions were mixed with deionized water at an oil volume fraction of 0.2. The resulting mixtures were sheared at 12,000 r/min for 5 min to generate coarse emulsions. Thereafter, 50 μL of the emulsion was added to 0.1% SDS solution (5 mL), and absorbance at 500 nm was recorded immediately (0 min) and 10 min after shearing. The SDS solution was measured as a control using the same protocol. The EAI and ESI were calculated using Equation (3) and Equation (4), respectively.

(3) EAI (m^2^·g^−1^) = 2 × T × A_0_ × n/1000 × C × φ

(4) ESI (%) = A_10_/A_0_ × 100

where T = 2.303 is a constant; C is the oil volume fraction (0.2); φ is the protein concentration (g/mL); and n is the dilution factor. At a wavelength of 500 nm, the absorbance at 0 min is represented by A_0_, and the absorbance at 10 min is designated as A_10_.

#### 2.6.2. Atomic Force Microscope (AFM)

After diluting the sample to 2 mol/g, 10 μL was dispensed onto silicon wafer surfaces. It was spread to form a homogeneous layer and dried in a desiccator at 25 °C for 1 min. Subsequently, the sample was analyzed using an Atomic Force Microscope (AFM, Bruker Dimension, Billerica, MA, USA). Scans were carried out in contact mode using a cantilever specified by a 3 N/m spring constant, 35 μm width, 225 μm length, and 2.8 μm thickness. The scan rate was set at 75 Hz per line.

#### 2.6.3. Analysis by Confocal Laser Scanning Microscopy (CLSM)

CLSM analysis was performed using an Olympus FV 1000 microscope (Olympus Corporation, Tokyo, Japan). AAFs were stained with Nile Red, whereas the OBG-CN complex was labeled with Nile Blue A solution.

### 2.7. Digestive Sustained Release and Cellular Uptake

#### 2.7.1. Digestive Sustained Release

The release of AAFs from OBG-AAF, CN-AAF, and CN-OBG-AAF was assessed using an in vitro digestion model, which covered oral, gastric, and small intestinal digestion phases. A 100 mL glass beaker was used to hold 20 mL of the original micelle’s solution in 10 mM phosphate buffer, which was then incubated at 37 °C for 2 min in a shaking incubator.

During the oral phase, at a pH of 6.8, 20 mL of simulated saliva fluid (SSF) with 0.03 g/mL α-amylase was added to the initial solution during the mouth stage. The mixture was then agitated at 100 rpm in a 37 °C incubator for 5 min to simulate oral mixing.

During the stomach phase, 20 mL of the solution was combined with 20 mL of simulated gastric fluid (SGF) containing pepsin and incubated at 37 °C. The pH of the SGF was adjusted to 3 using 0.1 M HCl, and the mixture was continuously stirred at 100 rpm in an incubator at 37 °C for 30 min to simulate stomach conditions.

During the small intestinal phase, 30 mL of the solution was relocated to a water-jacketed beaker, followed by the incorporation of 10 mL of simulated intestinal fluid (SIF). The SIF comprised 150 mM NaCl, 22.5 mM CaCl_2_, and 22.5 mg/mL bile extract, adjusted to a pH of 7. The mixture underwent a 10 min incubation before 5 mL of freshly prepared pancreatin solution (9.0 mg/mL) was introduced to facilitate lipolytic activity. A thermostatic water bath sustained the temperature at 37 °C, while the suspension was agitated at 250 rpm. Throughout the digestion process, samples were withdrawn at discrete time points between 0 and 120 min, and the released AAFs were quantified via HPLC/MS analysis.

#### 2.7.2. Cellular Uptake

Intestinal epithelial cells (IECs) were seeded into 6-well plates at a seeding density of 5 × 10^4^ cells/mL (2 mL per well) and cultivated in DMEM (pH of 7.4) supplemented with 10% heat-inactivated fetal bovine serum (FBS). The cells underwent incubation at 37 °C in a tissue culture incubator maintained at 5% CO_2_ and 95% relative humidity. The AAF micelles were stained with Nile Red fluorescent dye solution, and the DMEM solution was adjusted to a final concentration of 400 μg/mL. Following a 1 h treatment, the cells were fixed with 75% ethanol for 15 min, washed three times with Hanks’ solution, and stained with 4,6-diamidino-2-phenylindole (DAPI). The stained cells were then examined using an Olympus FV 1000 confocal laser scanning microscope (Olympus Corporation, Tokyo, Japan), with excitation wavelengths of 488 nm for Nile Red and 358 nm for DAPI. Images from individual optical planes and multiple serial optical sections were analyzed, and the images were scanned sequentially in both channels.

### 2.8. Statistical Analysis

One-way analysis of variance (ANOVA) was performed using SPSS 25.0 statistical software (SPSS Inc., Chicago, IL, USA). Duncan’s new multiple range test was employed to identify significant intergroup differences (*p* < 0.05).

## 3. Results and Discussion

### 3.1. Effects of Different Factors in UAE on Total Flavonoids

Based on the single-factor experimental design, an RSM with three factors and three levels was designed to optimize the extraction process in this study. The maximum extraction yield of flavonoids (2.18 ± 0.14%) was achieved at a 50% ethanol extraction concentration, a 40 mL/g liquid-to-material ratio, and 400 W ultrasonic extraction power (Figure S1A–D, Table S2). Flavonoids are considered one of the key bioactive components in AA [1]. Some studies have shown that extraction technology is closely correlated with the efficiency of flavonoid extraction [28]. In recent years, the UAE method has gained popularity due to its time-saving, efficient nature, simple operation, short extraction time, high efficiency, and minimal environmental impact. The use of ultrasonic waves promotes solvent penetration through thermal, mechanical, and cavitation effects, making flavonoid extraction more effective by aiding the dissolution of compounds into the solvent [29,30]. These variables play a crucial role in determining the extraction efficiency of plant bioactive compounds [31].

**Figure 1:**
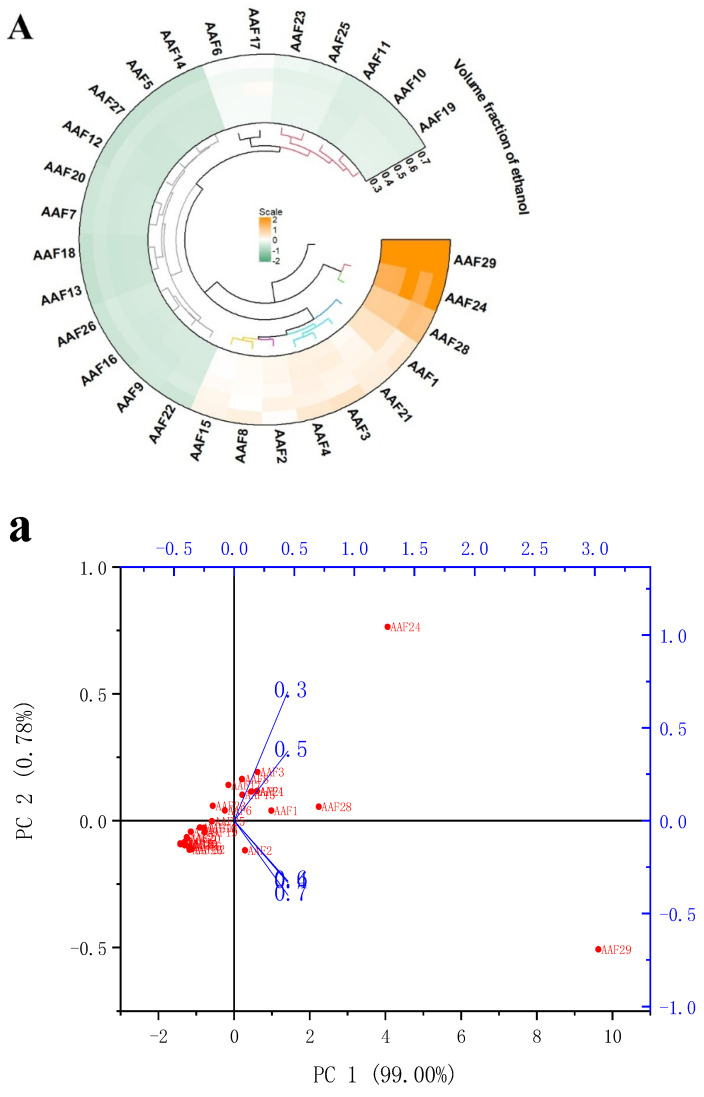

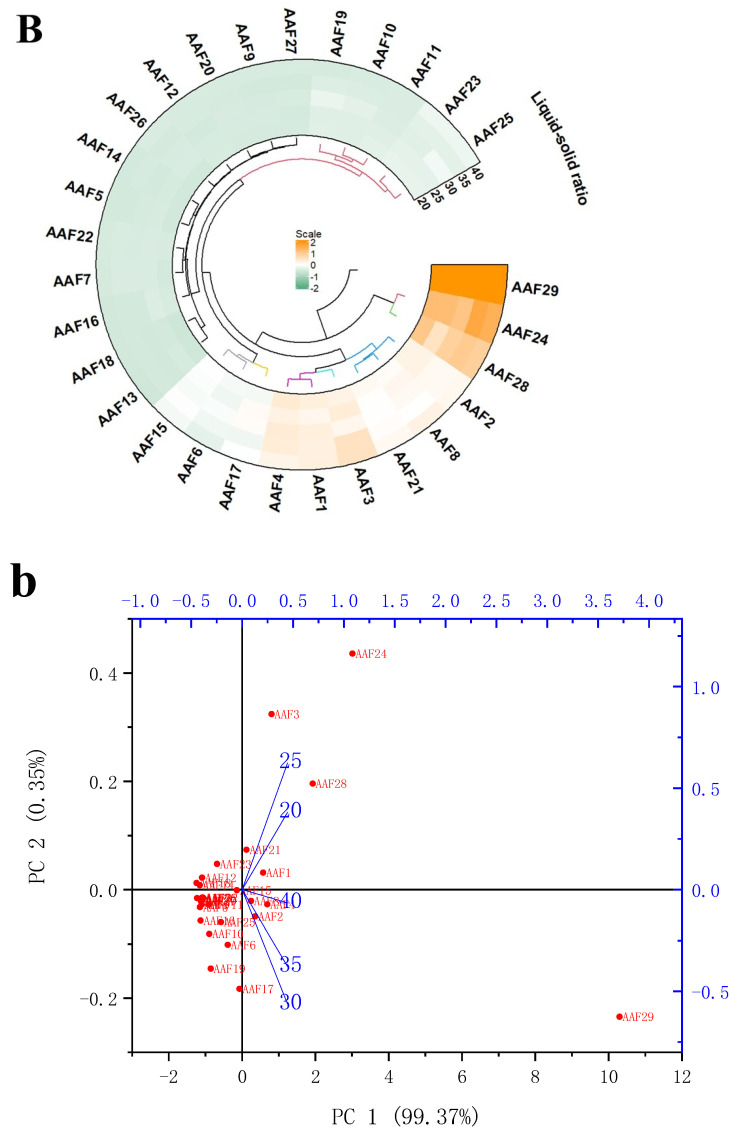

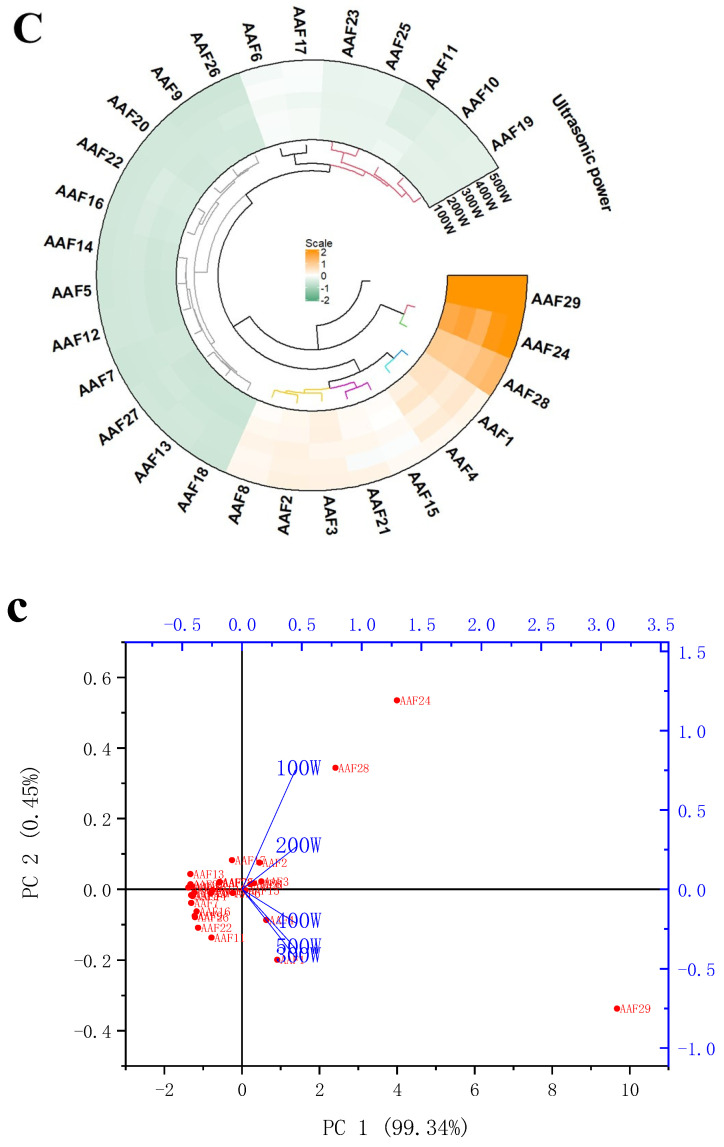
Hierarchical cluster analysis (HCA) and principal component analysis (PCA) between the AAF component and different extraction factors. (**A**) and (**a**), respectively, show the HCA heatmap and PCA score plot of the relationship between the AAF component and ethanol concentrations; (**B**) and (**b**) show the HCA heatmap and PCA score plot of the relationship between the AAF component and liquid to material ratio, respectively; (**C**) and (**c**) show HCA heatmap and PCA score plot of the relationship between the AAF component and ultrasonic power, respectively.

UPLC-MS was employed to identify flavonoids in AA from different factors. As presented in the negative ion mode, the peak emergence times of flavonoids ranged from 7.5 to 30 min, with the majority appearing between 13 and 20 min. The primary flavonoid components identified in AA included 29 compounds, numbered AAF 1 to AAF 29 (Table S3). The relationship between the AAFs and different extraction factors was subjected to multivariate analysis, including HCA heatmap (Figure 1A−C) and PCA score plot (Figure 1a−c). The HCA results showed that the positively correlations between extraction conditions (ethanol concentration, liquid-to-material ratio, and ultrasonic power) and flavonoid components included Rutin (AAF1), Kaempferol-3-O-rutinoside (AAF2), Isoquercitrin (AAF3), Isorhoifolin (AAF4), Kaempferol (AAF8), Kaempferol 7-neohesperidin (AAF15), Baicalin (AAF21), Isorhamnetin (AAF24), Hispidulin (AAF28), and 5,7,3′-trihydroxy-6,4′,5′-trimethoxy-flavonoids (AAF29).

As shown in Figure 1a−c, the first principal component (PC1, 99.00%, 99.37%, and 99.34%) and the second principal component (PC2, 0.78%, 0.35%, and 0.45%) accounted for the variation. Therefore, the information contained in PC1 can reflect the overall data characteristics of the samples [32]. Rutin (AAF1), Isoquercitrin (AAF3), Isorhoifolin (AAF4), Kaempferol 7- neohesperidin (AAF15), Baicalin (AAF21), Isorhamnetin (AAF24), and Hispidulin (AAF28) were on the positive axis of PC1, which may be related to extraction conditions involving 30% and 50% ethanol concentrations, 20 and 25 mL/g liquid to material ratios, and ultrasonic power of 100 W and 200 W. Kaempferol-3-O-rutinoside (AAF2), Centaurin-3, 5-di-o-glucoside (AAF14), and 5,7,3′-trihydroxy-6,4′,5′-trimethoxy-flavonoids (AAF29) were relatively closely grouped, indicating similar extraction conditions, including ethanol concentrations of 40, 60, and 70%, liquid to material ratios of 30 and 50 mL/g, and ultrasonic powers of 300, 400, and 500 W. The HCA results were consistent with the PCA results and suggest that the extraction factors of AAFs are highly variable, depending on the ethanol concentration, liquid/solid ratio, and ultrasonic power.

### 3.2. Construction of Glycosylation CN Micelles with Oat β-Glucan

**Figure 2:**
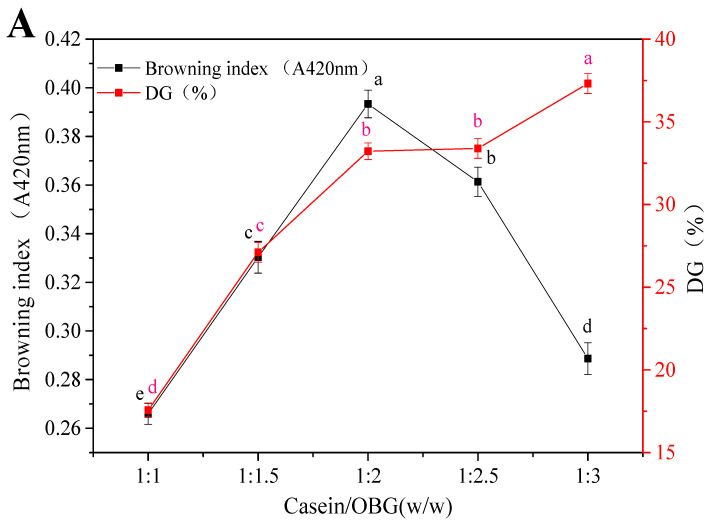

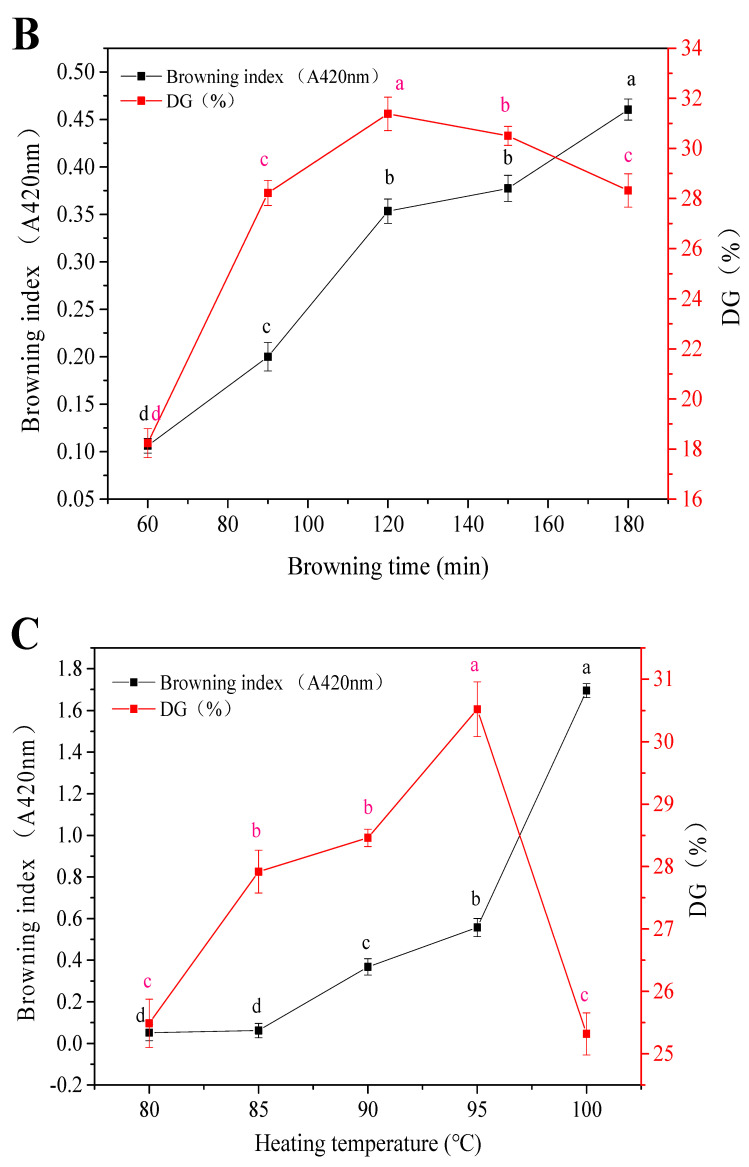

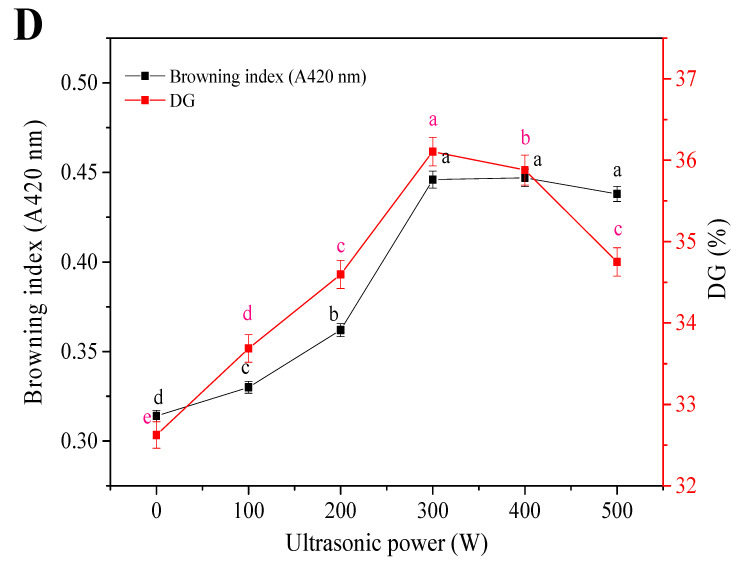
Effects of the addition ratio of CN and OBG (**A**), browning time (**B**), browning temperature (**C**), and ultrasonic power (**D**) on the degree of glycosylation. DG−the degree of grafting. The presence of the same letters indicates no significant differences (*p* > 0.05), whereas different letters denote significant differences (*p* < 0.05) in the figures.

The effect of different CN micelles and OBG addition ratios on the degree of glycosylation is shown in Figure 2. The degree of glycosylation can be characterized by DG between the amino and carbonyl groups, and the color of the reaction system is usually positively correlated with the course of the reaction. As shown in Figure 2A−C, with the increase in OBG, the viscosity of the solution increases and its fluidity decreases, resulting in the interaction between molecules becoming slower (Figure 2A). When the concentration is too high, a steric hindrance effect occurs, which hinders the grafting modification [33]. When the browning time was controlled for 120 min and the browning temperature was 95 °C, the browning index of the reaction increased first and then decreased with the increase in the reaction ratio, while the grafting degree increased gradually, reaching a maximum at a reaction ratio of 1:2 (Figure 2B,C). Previous studies have shown that when micellar casein and β-glucan coexist in the mixture, the compatibility of the biopolymer increases. In this study, as the temperature increases, CN micelles provide a larger volume for the dispersion of the β-glucan chains.

As shown in Figure 2D, within the ultrasonic intensity range of 0–300 W, the browning index of the reaction exhibits an upward trend with increasing pressure. The high ultrasonic intensity opens the structure of casein, increases the exposure of amino group on the protein chain, accelerates the degree of glycosylation reaction, promotes the accumulation of brown products, and leads to the browning index reaching its maximum at 300 W. With the extension of the treatment time, casein molecules may fold and aggregate, the process of glycosylation reaction may be blocked, or excessive accumulation of brown products hinders the grafting of protein and sugar molecules. Ultrasound processing can alter the protein conformation in secondary, tertiary, and quaternary structures by rupturing non-covalent interactions within protein molecules [34], depending on the intensity applied [35]. Native casein micelles consist of αs1-, αs2-, β-, and κ-casein, which are thermally unstable and their large structure is not conducive to the delivery and cellular uptake of nutrition components [6].

#### 3.2.1. FTIR Spectroscopy

**Figure 3:**
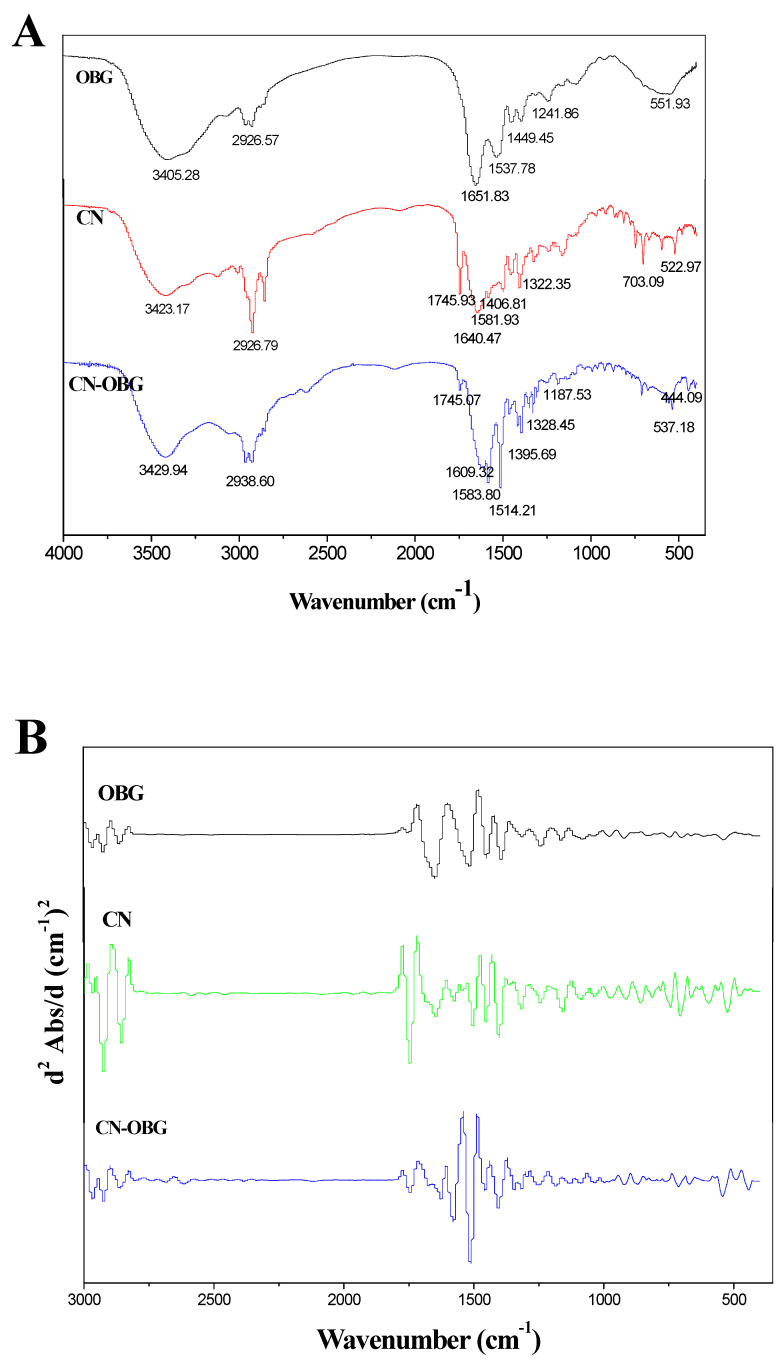

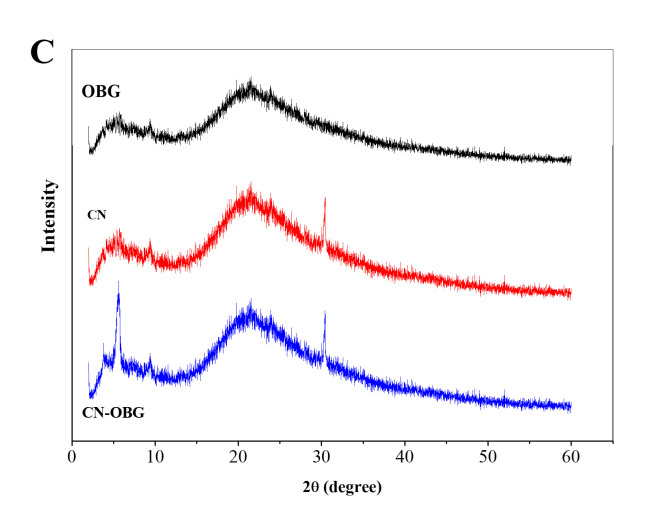

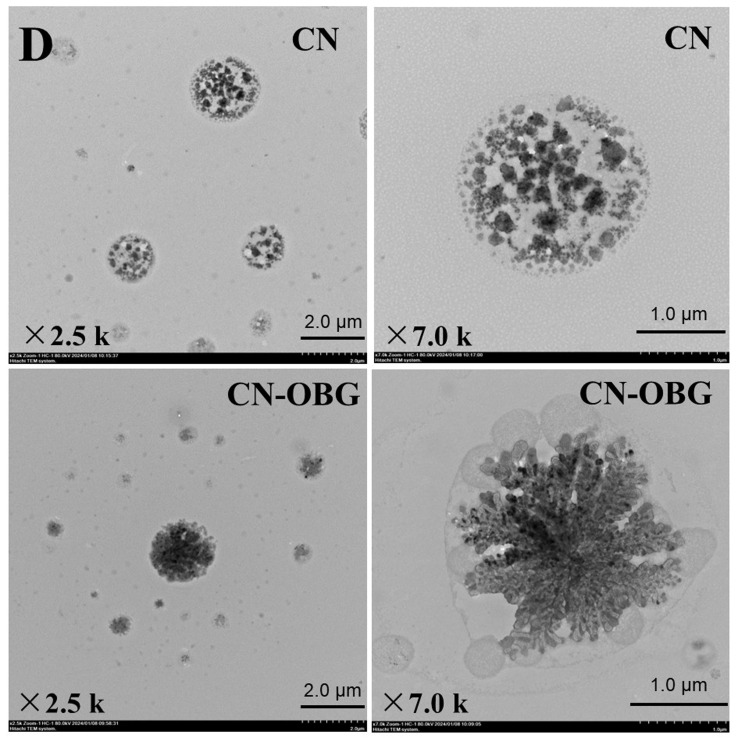
Fourier transform infrared (FTIR) analysis spectrum (**A**), corresponding second derivative spectra (**B**), X-ray diffraction (XRD) analysis (**C**), and transmission electron microscopy (TEM) image of CN and CN-OBG (**D**).

FTIR spectroscopy enables an in-depth analysis of the structural alterations in complexes. Functional groups in casein linked to Maillard reaction products—such as C=N, C=O, and C-N—might undergo alterations during the Maillard reaction. As shown in Figure 3A, major absorption peaks in OBG, CN, and CN-OBG micelles were observed at 3423 cm^−1^ (attributed to O-H stretching), 2926 cm^−1^ (assigned to C-H stretching and skewing), 1641 cm^−1^ (amide I, C=O stretching), 1542–1530 cm^−1^ (amide II, N−H bending), and 1100–1000 cm^−1^ (C-O-C glycoside bond stretching vibration). A broadening and a shift of the absorbance peak from 3405 cm^−1^ to 3429 cm^−1^ were observed in copolymers, suggesting that N-H and O-H groups of intermolecular hydrogen bonds underwent stretching and vibration after glycosylation [36]. Compared with CN, the CN-OBG micelles show a wide peak at around 1031 cm^−1^, and the absorption strength also increased to varying degrees, indicating that covalent cross-linking between casein and OBG caused side chain vibrations and protein structure changes, which may lead to more exposure of the hydrophobic groups [37]. In addition, the absorption peaks of glycosylated casein at 1654.37 cm^−1^ and 1546.72 cm^−1^ increased compared with that of normal pressure glycosylated casein, which might be due to the C=O stretching vibration and N-H bending vibration that occurred. However, the absorption strength of amide I and amide II bands decreased compared with untreated casein. It was proved that the ultrasonic treatment accelerated the glycosylation reaction.

Figure 3B illustrates the second derivative characteristics and secondary structure of the FTIR spectrum. The amide I band arises from the stretching of the carbonyl (C=O) group, while the amide II band results from N-H bending and C-N stretching vibrations. These various secondary structural components were identified based on the underlying bands within the amide I band. The identified FTIR bands corresponded to the following secondary structures: β-sheets (1610–1625 cm^−1^, 1630–1640 cm^−1^, 1670–1684 cm^−1^), random coil (1640–1648 cm^−1^), α-helices (1648–1658 cm^−1^), and β-turns (1660–1668 cm^−1^). Despite amino acid treatment, β-sheets remained the most abundant secondary structure. OBG increased the hydrophilicity of CN micelles through non-covalent bonding, driving a transition toward more ordered α-helical and β-sheet conformations. By binding to multiple hydrophobic sites on CN, OBG introduces additional hydrophilic groups such as –NH_2_, –OH, and –COOH. These groups facilitate hydration with water molecules in aqueous solutions, thereby improving the emulsification properties of the micelles.

#### 3.2.2. XRD Spectra

X-ray spectra can indicate the formation of complexes between organic ligands and metal ions, including the appearance, shifting, and disappearance of spectral features. As shown in Figure 3C, CN and CN-OBG had two sharp peaks at around 5.5° and 22.4°, which were caused by the main backbone crystalline network structure, indicating that CN was a crystalline polymer similar to the corn protein [38]. For OBG-bound CN, the intensity of diffraction peaks at 2θ = 31.4° showed a notable increase, indicating that chain associations significantly enhanced the scattering intensity. After CN was chelated with OBG, the spectroscopy of CN-OBG changed significantly. There were two strong and sharp diffraction peaks at around 5.5° and 31.4°, indicating that the CN-OBG was a different substance from CN with a new crystalline structure. The altered conformation, in which hydrophobic residues become increasingly solvent-exposed as a result of glycosylation, has significant implications for the protein structure and function [39]. This phenomenon indicates that the scattering intensity was significantly enhanced due to the formation of junction zones and chain.

#### 3.2.3. TEM

As shown in Figure 3D, the addition of OBG enhanced the co-assembly of CN, yet induced markedly distinct morphologies contingent on OBG concentration. Figure 3D showed that in the CN micelles, some of the smaller CN micelles showed inhomogeneous dispersion. However, in the presence of OBG, there were no large aggregates in the micelles, which was out of line with the OBG and glycosylation results. Furthermore, increasing the concentration of the cross-linking agent leads to a higher internal cross-link density within the CN. The number of covalent bonds in a single CN is sufficient to maintain its structural integrity. The more compact micelles obtained can also be attributed to the increased cross-link density. When an excess of OBG is used, cross-linking between micelles occurs, resulting in the formation of even smaller polymers. CN inherently contains hydrophobic regions, enabling it to effectively encapsulate diverse hydrophobic and hydrophilic compounds. Hydrophilic OBGs may localize to the micelle exterior and exhibit surface activity, attracting countercharges in polar water molecules and thereby forming electrical double layers [40]. Under water phase circumstances, the existence of electrical bilayers will make the micelles no longer adhere to each other and become a relatively stable colloidal solution.

### 3.3. Characterization of CN-OBG Micelles Loaded with Flavonoids

#### 3.3.1. AFM and CLSM

**Figure 4:**
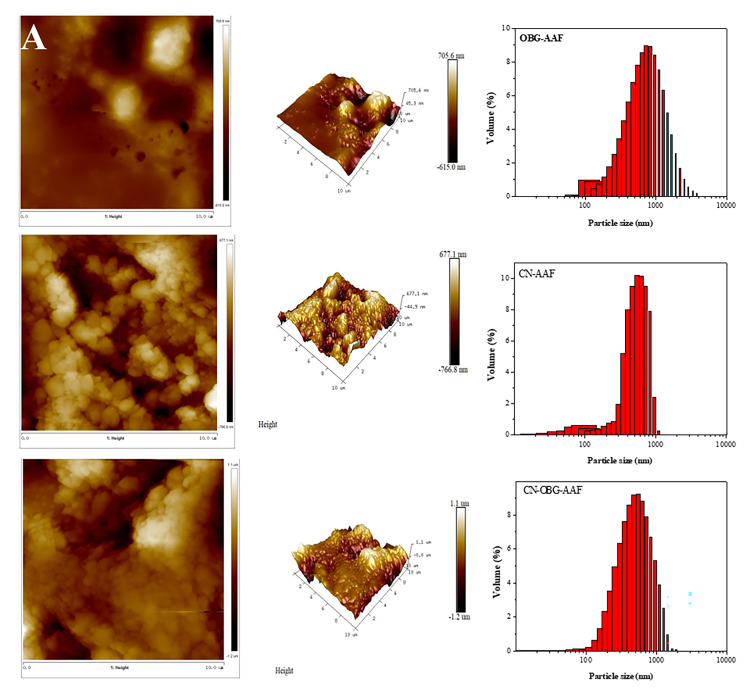

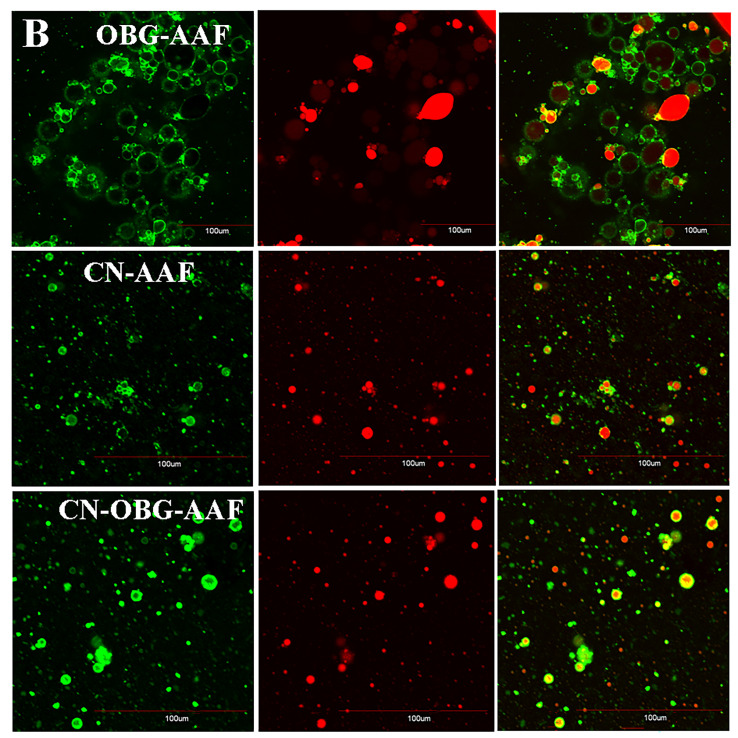
Atomic force microscope (AFM) and the particle size distribution images (**A**), and confocal laser scanning microscopy (CLSM) images (**B**) of AAFs loaded with OBG, CN, and CN-OBG. The pH is 6.0.

AFM serves as a powerful technique for examining molecules in their native state. The surface morphology and roughness of the CN, CN-FAA, and CN-OBG-AAF conjugates are shown in Figure 4A. The CN-FAA surface exhibited an overall smooth appearance, though interspersed with small, irregular particle agglomerations. These agglomerations primarily stemmed from intermolecular interactions driven by the presence of carboxyl, amino, and trace sulfhydryl groups in CN [41]. The analysis of CN-OBG conjugates revealed a neat surface and uniform shape. However, significant alterations in surface roughness and height were observed, likely resulting from the interaction between OBG and acidic amino acids on the micelles surface. When CN was pretreated with OBG, the particle size of the micelles decreased, and the particle size distribution became significantly narrower. In contrast, the particle size and distribution of OBG and CN alone were much broader. The presence of OBG on the CN surface was expected to influence the micelles’ zeta potential, possibly due to intermolecular forces, molecular surface tension, or intermolecular anisotropy. The glycosylation groups on CN were pivotal in binding bivalent minerals, acting as a primary site for bioavailability [42].

In Figure 4B, CLSM images showed that the AAFs stabilized with CN and CN-OBG had a uniform distribution. Conversely, larger FAA micelle droplets were detected in the OBG-AAF group. Red fluorescence was distinctly observable within the droplets, whereas green fluorescence was identified at the emulsion peripheries. This suggested that the AAFs were efficiently entrapped by CN and CN-OBG. A discernible layer of CN-OBG coated the AAF surface, appearing as a green, fluorescent closed ring. The hydrophobic properties of CN promoted the effective encapsulation of diverse hydrophobic and hydrophilic compounds. Situated on the micelle exterior, hydrophilic CN-OBG exhibited surface activity. In an aqueous milieu, these electrostatic double layers hindered micelle aggregation, thus sustaining a relatively stable colloidal solution.

#### 3.3.2. Load Rate of AAFs

**Figure 5:**
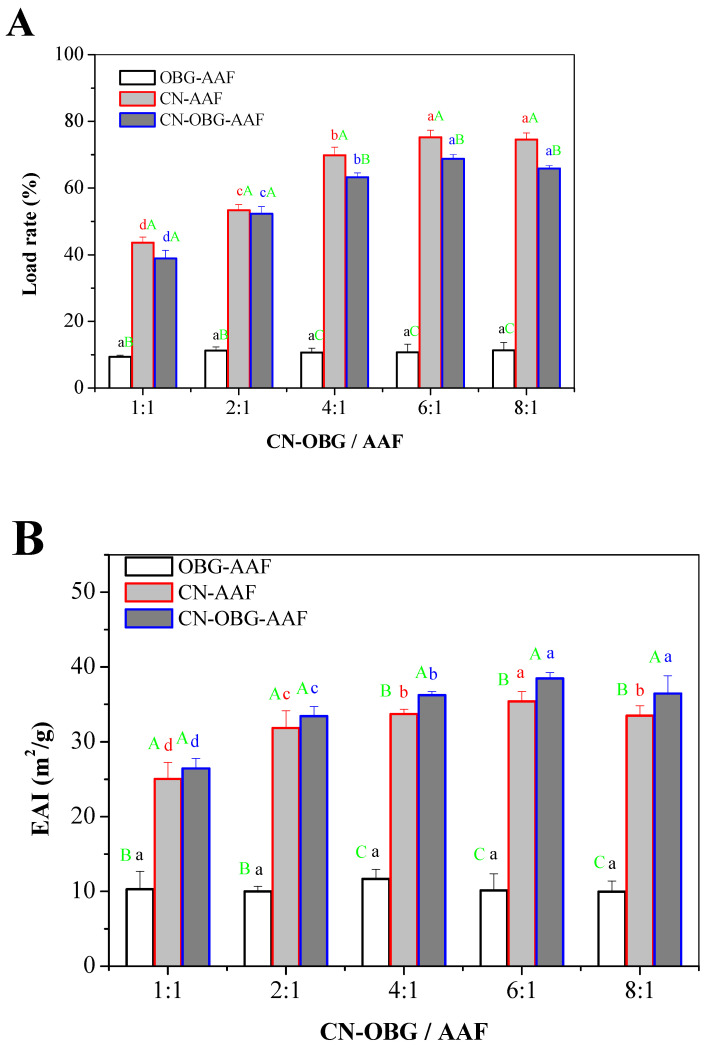

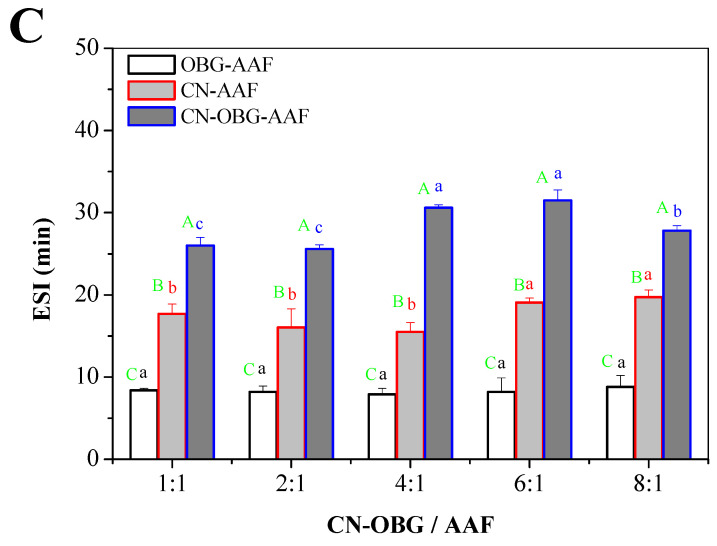
Load rate (**A**) of AAFs and emulsifying activity (EAI) (**B**), emulsifying stability (ESI) (**C**) of AAFs with different ratio between CN and OBG. In the figure, different capital letters indicate significant differences among OBG-AAF, CN-AAF, and CN-OBG-AAF (*p* < 0.05), while the same capital letters indicate no significant differences among OBG-AAF, CN-AAF, and CN-OBG-AAF (*p* > 0.05). Different lowercase letters in the figure indicate significant differences among different CN-OBG-AAF proportions within the same treatment (*p* < 0.05), while the same lowercase letters indicate no significant differences among different CN-OBG-AAF proportions within the same treatment (*p* > 0.05).

As depicted in Figure 5A, the emulsion’s encapsulation efficiency first increased and then decreased as the ratio of CN/CN-OBG to AAFs rose. Excessively high proportions of CN solution resulted in a reduced loading rate. At the outset, the disparities in loading rates were not substantial. In all groups, the encapsulation efficiency reached the maximum when the ratio of CN and CN-OBG to AAFs in the emulsion was 6:1. Additionally, the loading rate of crosslinked CN-OBG was notably higher than that of unmodified CN (*p* < 0.05). It shows that AAFs are not completely embedded in the phase when there is too much water, and the increase in emulsion viscosity due to the phase of too much oil is not conducive to the entrapment of oil phase [43]. Consequently, the droplets tended to coalesce, along with excess oil droplets, causing the particle size distribution to broaden and the emulsion to destabilize.

#### 3.3.3. Emulsion Stability

As depicted in Figure 5B, the emulsion’s emulsification activity gradually increased as the ratios of OBG, CN, and CN-OBG to AAFs rose, but declined once the CN:AAF ratio exceeded 6:1. The emulsification activity of CN-OBG was notably higher than other groups, suggesting that CN-OBG had the smallest droplet size and optimal emulsification performance. The EAI and ESI serve as effective metrics for evaluating the protein emulsifying properties at the oil–water interface [44]. A high EAI signifies strong oil–water adsorption capability, whereas a low EAI can stem from emulsion system instability. This instability promotes the formation of large aggregates that struggle to migrate to the oil–water interface [45]. This process forms a thinner interfacial film and stronger steric repulsion; thereby, improving the emulsifying stability of the AAF emulsion.

As illustrated in Figure 5C, the ESI of the CN-OBG group was significantly higher than that of the OBG and CN groups (*p* < 0.05). Compared with the OBG group, CN-OBG micelles enhanced the emulsion index by 25%. Meanwhile, the emulsion stability improved with the increasing amount of OBG added, likely due to the increased surface activity of the CN micelles. Governed by the ratio of polar to non-polar amino acids on the surface, micelle biopolymer surface activity is regulated by this compositional balance in protein biopolymers [46]. Owing to its highly flexible structure, casein was found to be ideal for emulsion stabilization. In nano-emulsions containing lactose and trehalose, CN has demonstrated superior emulsion stability at concentrations up to 10%, which can be attributed to the low separation rate and elevated viscosity [5]. The hydrophobic residues of CN provide steric-stabilizing properties around oil droplets in emulsions. Consequently, β-casein-stabilized emulsions exhibit a reduced tendency to flocculate and enhanced stability [47].

### 3.4. Digestion Stability In Vitro

**Figure 6:**
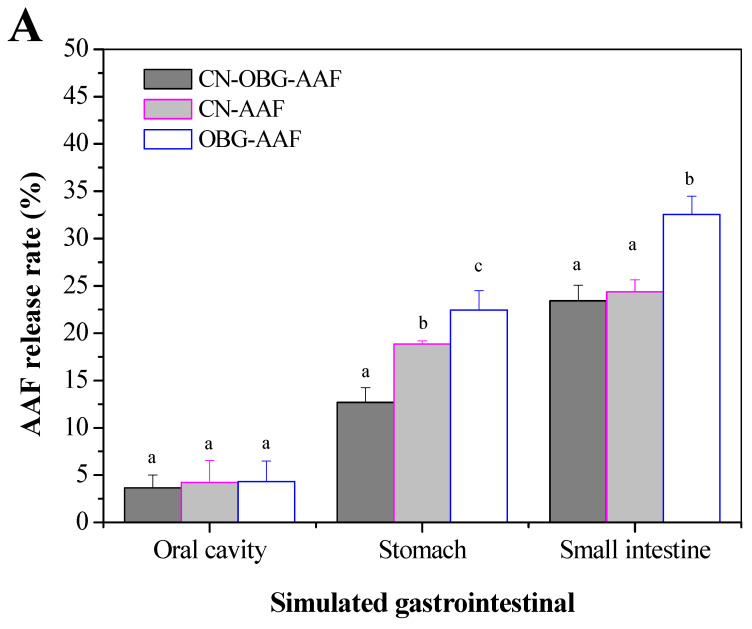

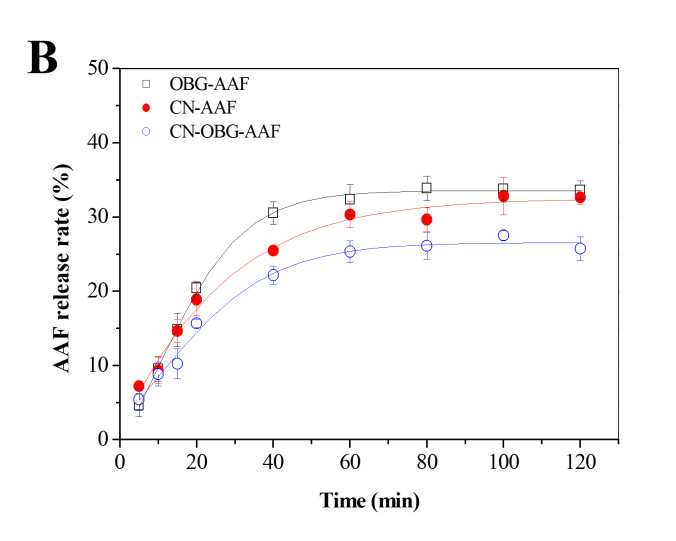

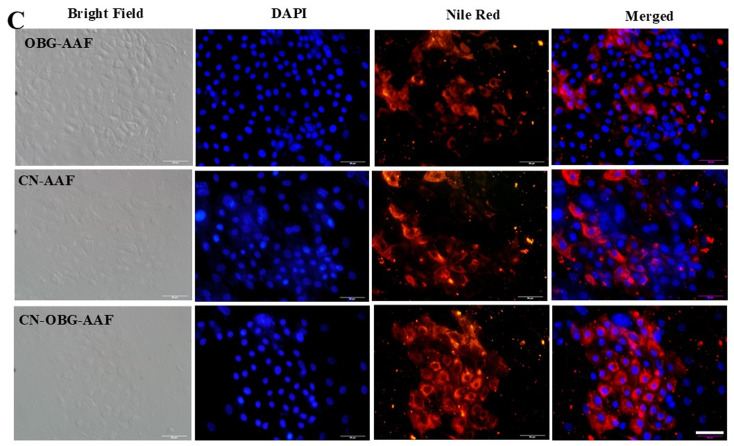
Digestion stabilization in vitro and cellular uptake images of OBG-AAF, CN-AAF, and CN-OBG-AAF in mouth, stomach, and small intestine, respectively. Bar = 50 μm. (**A**). Digestion stabilization in mouth, stomach, and small intestine (**B**). The relationship between AAF release rate and digestion time (**C**). Cellular uptake images of OBG-AAF, CN-AAF, and CN-OBG-AAF for the intestinal epithelial cells.

Figure 6 illustrates the AAF release profiles during simulated gastrointestinal digestion (mouth, stomach, and small intestine) of OBG-AAF, CN, and CN-OBG. As show in Figure 6A, the flavonoids in the three treatments are not digested in the mouth and have a low release rate. After 30 min of digestion, the release of AAFs remained relatively constant in OBG-AAF, CN, and CN-OBG treatment. During the simulated gastric and intestinal digestion, AAF release in the CN-OBG treatment showed a time-dependent decrease (Figure 6B). A rapid AAF release was observed within the first 40 min of digestion in the gastric and intestinal phases. Previous studies have shown that in the CN–OBG–AAF delivery system, OBG covalently grafted with CN creates a protective layer around AAFs, which prevents enzymatic degradation in the gastrointestinal environment and enables the slow release of AAFs [48]. In this study, the decreased release of AAFs might be attributed to the fact that the surface of AAF droplets with higher CN-OBG content is more resistant to lipase digestion compared to the surfaces of droplets in other emulsions. A recent investigation has shown that zein-polysaccharide nanoparticles can both enhance nutrient encapsulation efficiency and facilitate sustained release during in vitro digestion [49].

### 3.5. Cellular Uptake

The delivery of AAF-Nile Red into intestinal epithelial cells by CN-OBG, OBG, and the confocal laser scanning microscopy was used for the qualitative assessment of CN micelles. As illustrated in Figure 6C, the AAFs were stained with Nile Red fluorescent colorant, and AAF-Nile Red was efficiently transported into the intestinal epithelial cells in CN-OBG, OBG, and CN micelle treatments. More AAFs were successfully transformed into intestinal epithelial cells after 24 h of CN-OBG AAF treatment. Compared with CN-OBG AAF treatment, the uptake of OBG-AAF and CN-AAF treatment was lower. Nile Red fluorescence was clearly visible in cells incubated with AAF-loaded micelles, displaying a staining pattern matching that of free Nile Red-treated cells. As a control, CN micelles were additionally incubated with cells to exclude background fluorescence from CN-OBG micelles. Therefore, AAF-Nile Red was successfully delivered into the cells using CN-OBG micelles as a carrier. These results indicate that CN-OBG micelles can be internalized by intestinal epithelial cells and deliver their cargo in a controlled manner, as demonstrated by the in vitro release experiments.

The size and zeta potential of bioactive-loaded nanocarriers are critical parameters affecting colloidal stability, release kinetics, and intestinal wall absorption. The dimensions and charge of nanocarriers affect the uptake of biologically active loaded nanocarriers by the gastrointestinal tract through tight binding, internal circulation, and passive transport mechanisms [11]. The gastrointestinal uptake fate of maintenance oil droplets is mainly passive transport, while the absorption mode of nanoscale micelles is mainly transcellular transport. Therefore, the specific uptake of the AAFs loaded by CN-OBG micelles by intestinal epithelial cells was accelerated. Hydrophobic surface nanocarriers traverse the lipophilic bilayer of enterocyte cells more readily than their hydrophilic counterparts [50].

## 4. Conclusions

In summary, this study optimized the extraction yield of flavonoids using 50% ethanol, a liquid-to-solid ratio of 40 mL/g, and ultrasonic treatment at 400 W intensity. CN-OBG-AAF micelles were prepared with glycosylated casein as an emulsifier at a CN-to-OBG ratio of 1:2, with browning conditions set at 120 min and 95 °C. The CN-OBG micelles and glycosylation between OBG and CN significantly enhanced the emulsifying properties, digestive sustained release, and cellular uptake of AAF. This glycosylation modification is expected to serve as a cross-linking material for improving the emulsifying and delivery stability of self-assembled micelles for fat-soluble substances and pharmaceuticals.

## Data Availability

The original contributions presented in the study are included in the article/Supplementary Materials. Further inquiries can be directed to the corresponding authors.

## Supplementary Materials

The following supporting information can be downloaded at: https://www.mdpi.com/article/10.3390/foods14142435/s1, Figure S1: Effects of independent variables and Box–Behnken design (BBD) on the extraction of flavonoid compounds for AA. (A) Influence of ethanol concentration, (B) Influence of liquid-to-material ratio, (C) Influence of ultrasonic power, (D) Contour plots of liquid-solid ratio and ultrasonic power, (E) Contour plots of ethanol concentration and ultrasonic power, (F) Contour plots of ethanol concentration and liquid-solid ratio. Different letters between groups indicate significant differences (*p* < 0.05), while the same letter indicates no significant difference (*p* > 0.05). Table S1: Experimental design and results for RSM. Table S2: The models and predicted regression coefficients for response surface regression. Table S3: The main composition of AAF by UPLC/MS analysis.
