# Supplementary material for: Emulsifying Stability, Digestive Sustained Release, and Cellular Uptake of Alcohol-Soluble Artemisia argyi Flavonoids Were Improved by Glycosylation of Casein Micelles with Oat Glucan
Source: Foods. 2025 Jul 10;14(14):2435. doi: 10.3390/foods14142435 (PMC12295707; doi:10.3390/foods14142435)
Supplement: Supplementary file 1 [file foods-14-02435-s001.zip › Figure S1.pdf]

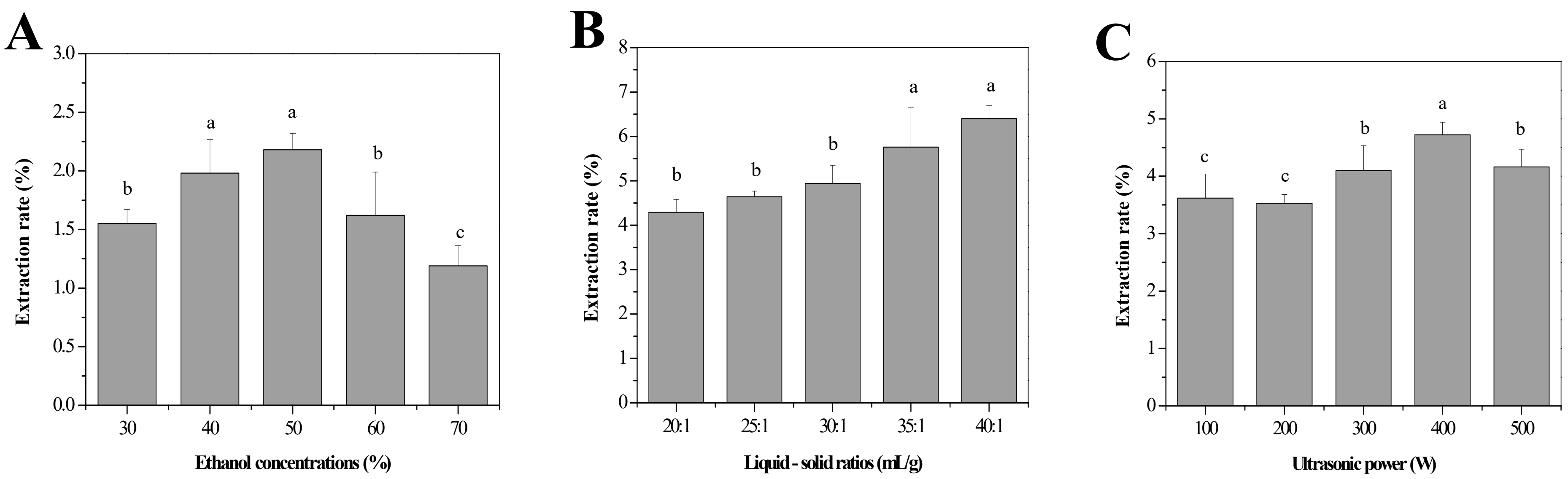
A
Extraction rate (%)
Ethanol concentrations (%)
30
40
50
60
70
B
Extraction rate (%)
Liquid - solid ratios (mL/g)
20:1
25:1
30:1
35:1
40:1
C
Extraction rate (%)
Ultrasonic power (W)
100
200
300
400
500

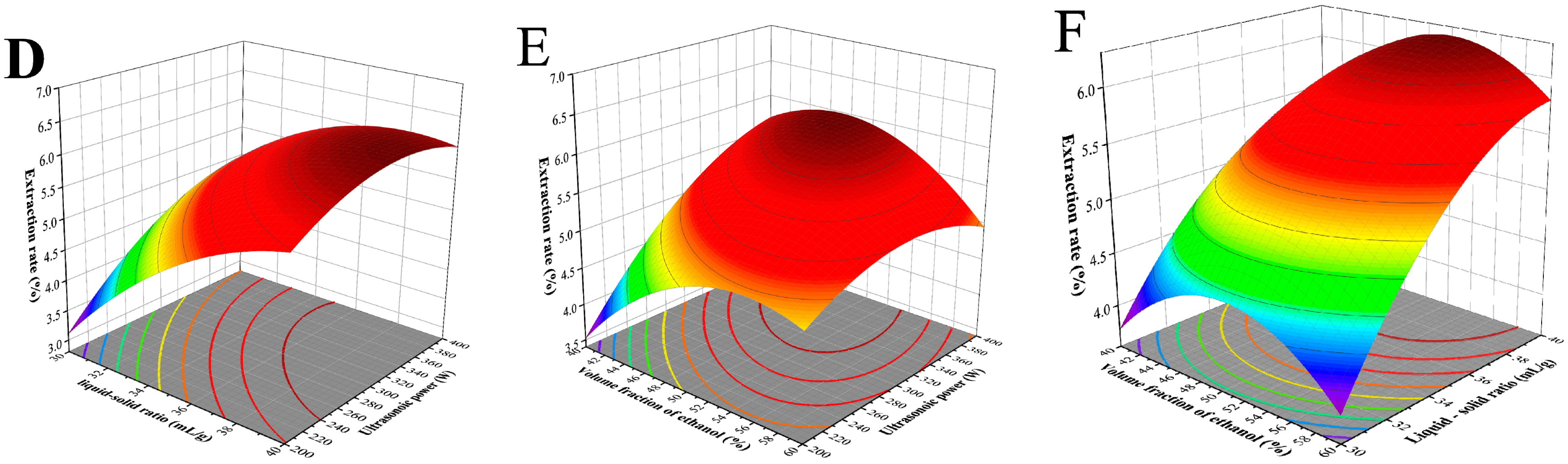


**Figure S1.** Effects of independent variables and Box–Behnken design (BBD) on the extraction of flavonoid compounds for AA. (A) Influence of ethanol concentration, (B) Influence of liquid-to-material ratio, (C) Influence of ultrasonic power, (D) Contour plots of liquid-solid ratio and ultrasonic power, (E) Contour plots of ethanol concentration and ultrasonic power, (F) Contour plots of ethanol concentration and liquid-solid ratio. Different letters between groups indicate significant differences ($P < 0.05$), while the same letter indicates no significant difference ($P > 0.05$).
