# Supplementary material for: Emulsifying Stability, Digestive Sustained Release, and Cellular Uptake of Alcohol-Soluble Artemisia argyi Flavonoids Were Improved by Glycosylation of Casein Micelles with Oat Glucan
Source: Foods. 2025 Jul 10;14(14):2435. doi: 10.3390/foods14142435 (PMC12295707; doi:10.3390/foods14142435)
Supplement: Supplementary file 1 [file foods-14-02435-s001.zip › Table S1.pdf]

Table S1. Experimental design and results for RSM.

Run	A	B	C	Extraction rate (%)
1	50	30	400	5.193
2	40	40	300	5.873
3	50	30	200	3.463
4	50	40	400	5.641
5	50	35	300	5.749
6	50	35	300	5.722
7	50	40	200	5.353
8	40	30	300	3.693
9	60	35	200	4.977
10	60	35	400	5.221
11	40	35	400	5.473
12	50	35	300	5.749
13	40	35	200	3.218
14	60	30	300	3.297
15	60	40	300	5.856
16	50	35	300	5.749
17	50	35	300	5.722

A. Ethanol concentration, %; B. Liquid - solid ratio, mL/g; C. Ultrasonic power, W.
