# Supplementary material for: Emulsifying Stability, Digestive Sustained Release, and Cellular Uptake of Alcohol-Soluble Artemisia argyi Flavonoids Were Improved by Glycosylation of Casein Micelles with Oat Glucan
Source: Foods. 2025 Jul 10;14(14):2435. doi: 10.3390/foods14142435 (PMC12295707; doi:10.3390/foods14142435)
Supplement: Supplementary file 1 [file foods-14-02435-s001.zip › Table S2.pdf]

Table S2. The models and predicted regression coefficients for response surface regression

Source	Sum of Squares	Df	Mean-Square	F-Value	*P*-Value	Significance
Model	13.95	9	1.55	8.96	0.0043	**
A- ethanol concentration	0.1496	1	0.1496	0.8646	0.0383	*
B- Liquid-solid ratio	6.26	1	6.26	36.18	0.0005	**
C-Ultrasonic temperature	2.55	1	2.55	14.74	0.0064	**
AB	0.0930	1	0.0930	4.77	0.0465	*
AC	1.01	1	1.01	5.84	0.0463	*
BC	0.5198	1	0.5198	3.00	0.0126	*
A^2	0.2041	1	0.2041	10.46	0.0060	**
B^2	0.7934	1	0.7934	4.59	0.0695	
C^2	0.6457	1	0.6457	3.73	0.0947	
Residual	1.21	7	0.1730			
Lack of Fit	1.21	3	0.4034	1844.72	< 0.0001	significant
Pure Error	0.0009	4	0.0002			
Cor Total	15.16	16				
R^2=0.9929						
R_{adj}^2=0.9859						
C V=3.08%						

Note：*P* value was applied to examine the significance of each coefficient. **indicates that the difference is extremely significant ($P < 0.01$); *Indicates that the difference is significant ($P < 0.05$).
