# Supplementary material for: Emulsifying Stability, Digestive Sustained Release, and Cellular Uptake of Alcohol-Soluble Artemisia argyi Flavonoids Were Improved by Glycosylation of Casein Micelles with Oat Glucan
Source: Foods. 2025 Jul 10;14(14):2435. doi: 10.3390/foods14142435 (PMC12295707; doi:10.3390/foods14142435)
Supplement: Supplementary file 1 [file foods-14-02435-s001.zip › Table S3.pdf]

Table S3. The main composition of AAF by UPLC/MS analysis.

Run No.	Retention time (min)	Molecular and ion peak	Peak area of flavonoid components of AA (mAU*min)	Molecular formula	Component name	Abb. code
1	13.58	$[M-H]^-$	539668	$C_{27}H_{30}O_{16}$	Rutin	AAF1
2	13.9	$[M-H]^-$	376688	C27$H_{30}O_{15}$	Kaempferol-3-O-rutinoside	AAF2
3	13.95	$[M-H]^-$	416254	$C_{21}H_{20}O_{12}$	Isoquercitrin	AAF3
4	15.18	$[M-H]^-$	428604	$C_{27}H_{30}O_{14}$	Isorhoifolin	AAF4
5	15.23	$[M-H]^-$	11622	$C_{21}H_{20}O_{11}$	Astragalin	AAF5
6	15.61	$[M-H]^-$	255521	$C_{21}H_{18}O_{11}$	Apigenin-7-O-glucuronide	AAF6
7	15.63	$[M-H]^-$	45952	$C_{21}H_{20}O_{10}$	Apigenin-7-O-glucoside	AAF7
8	18.6	$[M-H]^-$	339752	$C_{15}H_{10}O_6$	Kaempferol	AAF8
9	20.29	$[M-H]^-$	68351	$C_{15}H_{12}O_5$	Naringenin	AF9
10	20.62	$[M-H]^-$	148221	$C_{15}H_{10}O_5$	Apiin	AAF10
11	22.11	$[M-H]^-$	118530	$C_{17}H_{14}O_7$	Jaceosidin	AAF11
12	6.89	$[M+H]^+$	19148	$C_{27}H_{30}O_{17}$	Quercetin 3,4' -diglycoside	AAF12
13	7.66	$[M+H]^+$	8308	$C_{21}H_{20}O_{11}$	Galangin	AAF13
14	7.91	$[M+H]^+$	26118	$C_{27}H_{31}O_{16}$	Centaurin-3, 5-di-o-glucoside	AAF14
15	8.68	$[M+H]^+$	310223	$C_{27}H_{30}O_{15}$	Kaempferol 7-neohesperidin	AAF15
16	8.68	$[M+H]^+$	58103	$C_{15}H_{10}O_7$	Quercetin	AAF16
17	9.28	$[M+H]^+$	251747	$C_{21}H_{18}O_{12}$	Kaempferol glucuronide	AAF17
18	9.85	$[M+H]^+$	7542	$C_{15}H_{10}O_5$	Apigenin	AAF18
19	10.18	$[M+H]^+$	154894	$C_{22}H_{23}O_{11}$	Paeoniflorin 3-O-β-D-glucopyranoside	AAF19
20	10.18	$[M+H]^+$	47528	$C_{22}H_{22}O_{12}$	Isorhamnetin 3-galactoside	AAF20
21	10.31	$[M+H]^+$	379256	$C_{21}H_{18}O_{11}$	Baicalin	AAF21

22	11.45	$[M+H]^+$	74439	$C_{15}H_{12}O_6$	Eriodictyol	AAF22
23	12.03	$[M+H]^+$	185933	$C_{15}H_{10}O_6$	Galuteolin	AAF23
24	12.31	$[M+H]^+$	1035740	$C_{16}H_{12}O_7$	Isorhamnetin	AAF24
25	12.89	$[M+H]^+$	189389	$C_{17}H_{14}O_7$	3, 7-dimethoxy-3 ',4', 5-trihydroxyflavones	AAF25
26	12.97	$[M+H]^+$	67218	$C_{17}H_{14}O_8$	3',5,5', 7-tetrahydroxy-4 ', 6-dimethoxy-flavone	AAF26
27	13.49	$[M+H]^+$	21329	$C_{16}H_{14}O_6$	Aurantiamarin	AAF27
28	13.96	$[M+H]^+$	745741	$C_{16}H_{12}O_6$	Hispidulin	AAF28
29	14.94	$[M+H]^+$	2248095	$C_{18}H_{16}O_8$	5,7,3' -trihydroxy-6,4',5' -trimethoxy-flavonoids	AAF29
